# Tuina for low back pain: Protocol for a systematic review and meta-analysis

**DOI:** 10.1097/MD.0000000000011979

**Published:** 2018-08-24

**Authors:** Zhiyong Fan, Qiang Tian, Rusong Guo, Yu Zhang, Shan Wu

**Affiliations:** a The Second Affiliated Hospital of Guangzhou University of Chinese Medicine, Guangdong Provincial Hospital of Chinese Medicine; b The Second Clinical School of Guangzhou University of Chinese Medicine, Guangzhou, China.

**Keywords:** low back pain, protocol, systematic review, tuina

## Abstract

**Background:**

Low back pain (LBP) is one of the most common symptoms prompting patients to seek treatment. In China, tuina is a common treatment for LBP, but its effects and safety remain uncertain. This protocol is to provide the methods used to assess the effectiveness and safety of tuina for the treatment of patients with LBP.

**Methods:**

We will search the following databases by electronic methods: MEDLINE, PUBMED, EMBASE, CINAHL, the Chinese Biomedical Literature Database (CBM), the China National Knowledge Infrastructure (CNKI), Wanfang Data (WAN FANG), and VIP Information (VIP). The time limit for retrieving studies is set to be built in and before July 2018 for each database. The therapeutic effects according to the Visual Analog Scale (VAS) will be accepted as the primary outcomes. We will use RevMan V.5.3 software as well to compute the data synthesis carefully when a meta-analysis is allowed.

**Results:**

This study will provide a high-quality synthesis of current evidence of tuina for the treatment of patients with LBP.

**Conclusion:**

The conclusion of our systematic review will provide evidence to judge whether tuina is an effective intervention for patient with LBP.

**PROSPERO registration number:**

PROSPERO CRD 42018096762.

## Abstract 2

Supplemental Digital Content is available in the text

## 1 Introduction

Low back pain (LBP) is one of the most common symptoms prompting patients to seek treatment. The lifetime prevalence of LBP is estimated to be over 50%.^[1]^ The US National Health Interview Survey in 2002 showed that 26% of Americans had LBP for at least 1 day in the last 3 months.^[2]^ A survey based on the United Kingdom population showed that the prevalence of LBP in the population over a 1-month period ranged from 35% to 37%.^[3,4]^

The prevalence of LBP varies widely across countries around the world, with annual rates typically ranging from 4% to 93%.^[5]^ The recurrence of LBP is more common, as the rate of acute LBP turning into recurrent or chronic LBP within 1 year is 35% to 75%, while a significant proportion of patients with persistent pain give up seeking further medical treatment.^[4]^

LBP can be caused by specific pathological conditions such as infections, tumors, fractures, and inflammation. However, the pain of 85% of patients is non-specific, which suggests that the pain cannot be attributed to one of the above defined conditions, but rather to some ambiguous cause.^[6]^ The main purpose of LBP treatment is to relieve pain and restore function.^[7]^ Treatment begins with the patient education and self-care guidance before considering the use of treatments that have been proven effective by evidence-based medicine, including relieving pain and muscle spasms through drug therapy and physical therapy. Among them, patient education is the basis for the treatment of LBP.^[8]^ Although these traditional treatments have proven some efficacy, these treatments are not always effective and even have some serious side effects.^[9]^

Therefore, in order to find more effective treatments, many people have turned their attention to other treatments, such as complementary and alternative medicine (CAM). Although CAM itself has some side effects, in view of the numerous treatments of CAM and the positive effects to a certain extent, more and more researchers are focusing on various CAM therapies such as massage and acupuncture.^[10]^ Obviously, as a kind of traditional Chinese medicine, acupuncture has shown considerable efficacy on the treatment of pain, and is accepted worldwide.^[11]^

Tuina, a non-drug natural therapy as well as physical therapy, normally means that the practitioner uses his own hands to apply continuous pressure to the patient's body surface, injured parts, discomfort, specific acupoints, and painful parts to treat the disease. It combines many principles of acupuncture, including the use of acupuncture points.^[12,13]^ Tuina has been practiced in China for thousands of years. It is a highly regarded treatment and is known to have a certain therapeutic effect on various diseases, including the treatment of LBP.^[14,15]^

However, due to the prevalence of insufficient sample size, low methodological quality and lack of high-quality research in relevant clinical trials, the effectiveness and safety of tuina for LBP remains controversial.^[16]^ Nevertheless, to the best of our knowledge, although there are a large number of clinical reports on tuina treatment for LBP, there is a lack of systematic review or meta-analysis of its efficacy. Therefore, this study applies the method of evidence-based medicine to analyze and assess the global clinical randomized controlled trials of tuina for LBP, to provide better evidence for further study of the clinical efficacy of tuina for LBP. This study will try to solve the following problems. Is tuina a safe and effective treatment for LBP? What problems exist in the relevant clinical research at present and provide good suggestions for future research design.

## 2 Methods

### 2.1 Inclusion criteria for study selection

#### 2.1.1 Types of studies

We will include randomized controlled trials (RCTs) of tuina for LBP in the treatment groups.

#### 2.1.2 Types of patients

We will include adult patients with LBP (> 18 years old). The duration of LBP is not limited and includes patients with (sub) acute (=12 weeks) or chronic LBP (> 12 weeks). Patients with LBP caused by infection, metastatic disease, tumor or fracture are excluded. Patients with LBP associated with pregnancy and childbirth are also excluded.

#### 2.1.3 Types of interventions

The experimental group receives tuina treatment, while the control group adopts treatments generally approved for treating LBP, such as oral medication, physical therapy, behavioral therapy or acupuncture, and so on.

#### 2.1.4 Types of outcome measures

##### 2.1.4.1 Primary outcomes

LBP will be assessed by the Visual Analog Scale (VAS) (0–100)^[17]^ or the 11-point Numeric Rating Scale (NRS).^[18]^

##### 2.1.4.2 Secondary outcomes

- Quality of life will be measured by the 36-item Short-Form Health Survey (SF-36).^[19]^
- Adverse events.

### 2.2 Search methods for the identification of studies

#### 2.2.1 Electronic searches

We will search the following databases by electronic methods: MEDLINE, PUBMED, EMBASE, CINAHL, the Chinese Biomedical Literature Database (CBM), the China National Knowledge Infrastructure (CNKI), Wanfang Data (WAN FANG), and VIP Information (VIP). The time limit for retrieving studies is set to be built in and before July 2018 for each database. We will also retrieve unpublished protocols and summary results by searching the clinical trial registry at https://clinicaltrials.gov/. After discussing with all reviewers, a temporary search strategy has been identified. Keywords include “tuina”, “low back pain” and “randomized controlled trial“. The search strategy for PUBMED is shown in Appendix A. The search terms used in the Chinese database have the same meaning as the terms used in the English database.

#### 2.2.2 Searching other resources

The following methods are also used to find potential studies in compliance with the criteria.

Search:

- Previously published reviews related to tuina for LBP.
- Meeting abstracts may contain ongoing or unpublished trials related to tuina for LBP. If applicable, we will contact the author and collect relevant data.

### 2.3 Data collection and analysis

#### 2.3.1 Selection of studies

**Figure 1:**
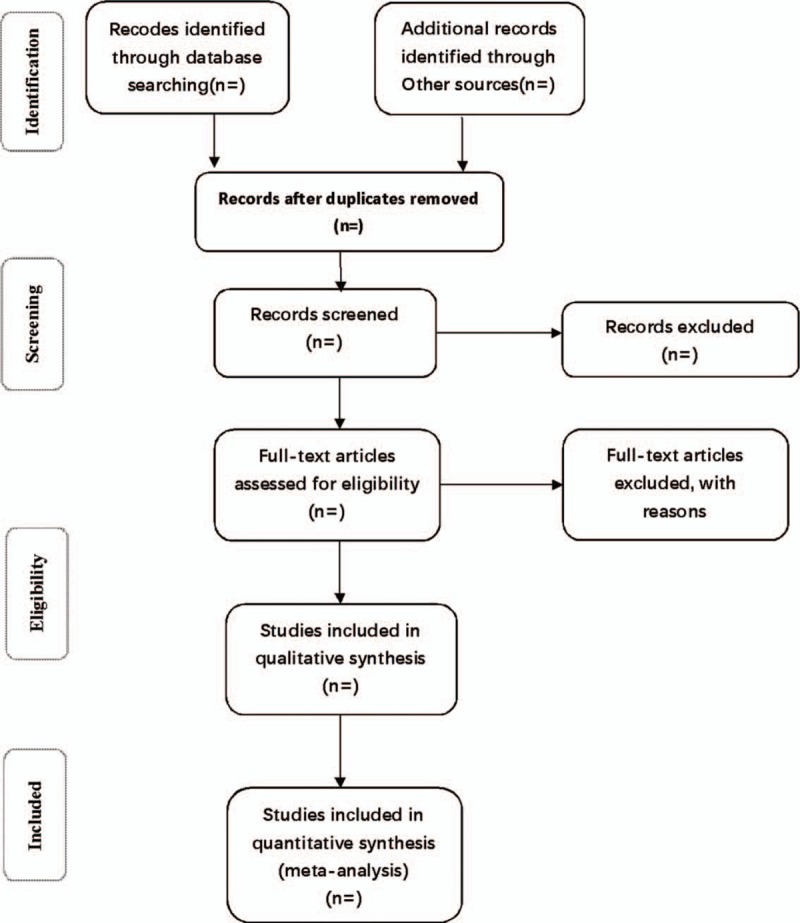
Flow diagram of study selection process.

Researchers will discuss and determine screening criteria within the group before searching the studies. First, the studies searched from the electronic database and from other sources are imported into the literature management system of EndnoteX7 for duplicate removal. Then, the 2 researchers will independently exclude clearly unqualified studies by reading the headings and abstracts, and then read the full text, discuss within the group and contact the author to understand the details of the studies to determine the final included studies. Once any disagreement occurs during the screening process, it will be resolved through discussion and consensus between the 2 researchers or by consulting a third party arbitrator. The entire process of study selection is summarized in the PRISMA flow diagram (Fig. 1).

#### 2.3.2 Data collection and management

The 2 researchers independently used the data extraction tables discussed in advance to extract data from the included studies. The extracted data will include basic information about the study (eg, author, year of publication, country), basic patient information (eg diagnostic criteria, age, gender, duration of illness, and so on), interventions (eg, parts of tuina, course of treatment), outcome indicators and other project data (eg, funding sources and ethical approvals). Any disagreement on data extraction will be resolved through discussions or negotiations with the third arbitrator. If the data provided in the research is unclear, missing or presented in a form that is not extractable or difficult to extract reliably, we will contact the author of the research for clarification.

#### 2.3.3 Assessment of risk of bias in included studies

The quality assessment for each RCT will be assessed independently by 2 reviewers using the Cochrane Collaboration Risk of Bias Tool checklist. The tool assesses the methodological quality from seven aspects: random sequence generation, allocation concealment, blinding of participants and personnel, blinding of outcome assessment, incomplete outcome data, selective reporting, and other bias. Considering these areas, each trial will be divided into low risk, high risk and ambiguous risk. Any disagreement will be discussed with the third author to achieve consensus.

#### 2.3.4 Measures of treatment effect

For continuous data (eg, VIS, NRS, and SF-36 scores), the mean difference (MD) and the corresponding 95% confidence interval (CIs) will be used. In addition, we will use standardized mean differences (SMDs) if necessary. For dichotomous data (eg, number of patients during trial follow-up and adverse events), we will use the risk ratio (RR) and the corresponding 95% CIs. Other dichotomous data will be converted to RR values.

#### 2.3.5 Dealing with missing data

We will contact the authors of the studies included in the research to try to obtain any missing information from their trials. If data is not available, the study will not be included in the data analysis.

#### 2.3.6 Assessment of heterogeneity

Prior to data analysis, the *χ*^*2*^ test will be used to determine the homogeneity of the study. If the resulting *P*-value exceeds 0.1, indicating significant heterogeneity in the trial, the cause of the heterogeneity will be analyzed and a sub-group analysis will be performed.

#### 2.3.7 Assessment of reporting bias

If sufficient research is included (at least 10 trials), a funnel plot will be constructed to assess publication bias.

#### 2.3.8 Data synthesis

Meta-analysis will be performed using RevMan 5.3 software (The Cochrane Collaboration, Oxford, England). The heterogeneity among the results of each included study will be tested using the *χ*^*2*^ test. When there is statistical homogeneity among the results (*P* > .1), the fixed effect model will be used for meta-analysis; if there is statistical heterogeneity among the results (*P* ≤ .1), the heterogeneity source will be analyzed, and subgroup analysis will be performed based on factors that may lead to heterogeneity. When there are sufficient similarities among the subgroup results (subgroup *P* > .1), a fixed effect model will be used for meta-analysis; if there is statistical heterogeneity between the subgroups in the study instead of clinical heterogeneity or the difference is not statistically significant, a random effect model will be used for meta-analysis; if the heterogeneity between the groups is too large, a descriptive analysis will be performed.

#### 2.3.9 Subgroup analysis

Subgroup analysis will be performed to assess the heterogeneity of the research:

Clinical consideration

- Different acupuncture points for tuina
- Different types of LBP (eg, acute and chronic)

Methodological consideration

- Trials with ambiguous or high bias risks.

#### 2.3.10 Sensitivity analysis

Sensitivity analysis is an important method primarily used to assess the robustness and reliability of the combined results of meta-analysis. It is a commonly used sensitivity analysis method to eliminate each of the included studies before combining the effect quantities or to combine the effect quantities after changing the inclusion and exclusion criteria or eliminate certain types of studies. After the quality assessment of the included literature, if there are possible low-quality studies, sensitivity analysis will be required.

#### 2.3.11 Ethics and dissemination

This systematic review does not need ethical approval because there are no data used in our study that are linked to individual patient data. In addition, the findings will be disseminated through a peer-review publication.

## 3 Discussion

LBP causes severe pain to individuals, but most currently available treatments are not sufficient to control pain.^[20]^ Pharmacological methods have associated side effects, and surgery is expensive and not suitable for every patient.^[9]^ Tuina has been used in China for thousands of years and is generally considered to be a safe and effective measure to alleviate pain.^[21]^ However, when the effectiveness of tuina for LBP is still unclear, it is difficult for clinicians to make appropriate recommendations. This is a protocol for systematic review to assess the safety and effectiveness of tuina for LBP. Since there was no systematic review of tuina for LBP before, we hope that this systematic review will help clinicians make decisions in practice and promote the progress of tuina research.

However, there are some potential limitations in this research. Different forms of tuina and different levels of methodological quality included in the trial may result in significant heterogeneity. There may also be a lack of some related studies, as it only includes English and Chinese studies.

## Author contributions

**Conceptualization:** Zhiyong Fan, Shan Wu.

**Data curation:** Zhiyong Fan, Qiang Tian, Yu Zhang, Shan Wu.

**Formal analysis:** Qiang Tian.

**Funding acquisition:** Qiang Tian.

**Project administration:** Rusong Guo.

**Resources:** Rusong Guo.

**Writing – original draft:** Zhiyong Fan, Yu Zhang, Shan Wu.

**Writing – review & editing:** Zhiyong Fan, Shan Wu.

## Supplementary Material

Supplemental Digital Content
