# Supplementary material for: Tuina for low back pain: Protocol for a systematic review and meta-analysis
Source: Medicine (Baltimore). 2018 Aug 24;97(34):e11979. doi: 10.1097/MD.0000000000011979 (PMC6113039; doi:10.1097/MD.0000000000011979)
Supplement: Supplemental Digital Content [file medi-97-e11979-s001.docx]

**Appendix A.**

***Search strategy used in PubMed database***

#1 Low Back Pain OR Back Pain

#2 Tuina OR Massage

#3 Randomized controlled trial OR clinical study OR Clin-ical Trial OR Controlled study OR Controlled Trial OR Random*Control* study OR random* Control* Trial

#1 AND #2 AND #3
